# Improving spatial prioritisation for remote marine regions: optimising biodiversity conservation and sustainable development trade-offs

**DOI:** 10.1038/srep32029

**Published:** 2016-08-24

**Authors:** Cordelia H. Moore, Ben T. Radford, Hugh P. Possingham, Andrew J. Heyward, Romola R. Stewart, Matthew E. Watts, Jim Prescott, Stephen J. Newman, Euan S. Harvey, Rebecca Fisher, Clay W. Bryce, Ryan J. Lowe, Oliver Berry, Alexis Espinosa-Gayosso, Errol Sporer, Thor Saunders

**Affiliations:** 1 Department of Environment and Agriculture, Curtin University, Bentley Campus, Perth, WA 6102, Australia; 2 Australian Institute of Marine Science, UWA Oceans Institute (M096), 35 Stirling Highway, Crawley, Perth, WA 6009, Australia; 3 Western Australian Fisheries and Marine Research Laboratories, Department of Fisheries, Government of Western Australia, P.O. Box 20, North Beach, WA, 6920, Australia; 4 School of Earth and Environment and the UWA Oceans Institute, The University of Western Australia, 35 Stirling Highway, Crawley, WA 6009, Australia; 5 CSIRO Oceans and Atmosphere Flagship, PMB 5, Floreat, Western Australia, 6014, Australia; 6 ARC Centre of Excellence for Environmental Decisions, The University of Queensland, Brisbane, QLD 4072, Australia; 7 Oceans Institute, University of Western Australia, Crawley, WA 6009, Australia; 8 Australian Fisheries Management Authority, Darwin, NT 0801, Australia; 9 Western Australian Museum, Perth, WA 6986, Australia; 10 ARC Centre of Excellence for Coral Reef Studies, University of Western Australia, Crawley, Australia; 11 Civil, Environmental and Mining Engineering and the UWA Oceans Institute, University of Western Australia, Crawley, WA 6009, Australia; 12 Department of Primary Industry and Fisheries, Darwin, NT 0801, Australia

## Abstract

Creating large conservation zones in remote areas, with less intense stakeholder overlap and limited environmental information, requires periodic review to ensure zonation mitigates primary threats and fill gaps in representation, while achieving conservation targets. Follow-up reviews can utilise improved methods and data, potentially identifying new planning options yielding a desirable balance between stakeholder interests. This research explored a marine zoning system in north-west Australia–a biodiverse area with poorly documented biota. Although remote, it is economically significant (i.e. petroleum extraction and fishing). Stakeholder engagement was used to source the best available biodiversity and socio-economic data and advanced spatial analyses produced 765 high resolution data layers, including 674 species distributions representing 119 families. Gap analysis revealed the current proposed zoning system as inadequate, with 98.2% of species below the Convention on Biological Diversity 10% representation targets. A systematic conservation planning algorithm Maxan provided zoning options to meet representation targets while balancing this with industry interests. Resulting scenarios revealed that conservation targets could be met with minimal impacts on petroleum and fishing industries, with estimated losses of 4.9% and 7.2% respectively. The approach addressed important knowledge gaps and provided a powerful and transparent method to reconcile industry interests with marine conservation.

Protected areas are internationally recognised as an effective tool for biodiversity conservation^1^,^2^,^3^. A protected area is a ‘clearly defined geographical space, recognised, dedicated and managed, through legal or other effective means, to achieve the long term conservation of nature with associated ecosystem services and cultural values’ (IUCN definition 2008). The benefits of large-scale networks of protected areas to conserve biodiversity, maintain and improve ecosystem health and resilience, and to help ensure the sustainability of natural resources are well documented^2^,^4^,^5^,^6^. In 2002, the Convention on Biological Diversity (CBD) called for at least 10% of each of the world’s terrestrial and marine ecoregions to be effectively conserved by 2010. Globally, protected areas cover 14% of the terrestrial environment, but less than 3.4% of the marine environment^7^. In response, the CBD retained the 10% target for the marine environment with a revised achievement date of 2020^8^. This has focussed attention on the need to fill significant gaps in representation of the marine realm.

A number of studies highlight the need for establishing protected areas in locations where they address primary threats and gaps in representation, not simply where they can minimise conflict^9^,^10^,^11^. However, some systematic analyses reveal that minimising conflict between stakeholders with different objectives is often the prevailing driver of protected area location^9^,^12^,^13^. For example, Klein *et al*.^11^ in a global synthesis examining the range of 17,348 marine species found 97.4% had <10% of their ranges represented within stricter conservation classes. In addition, Devillers *et al*.^10^ reviewed the global pattern in marine protected areas (MPAs) finding an increasing trend for large MPAs to be placed in remote and unpromising areas for commercial use. They suggested that very large MPAs (>100,000 km^2^), such as Natural Park of the Coral Sea (France), Chagos (UK) and Coral Sea (Australia), have been declared in an effort to meet international conservation targets while minimising conflict. Each park covers about 10% of the EEZ of their respective country. However, the effectiveness of extensive, remote protected areas for conserving biodiversity is unclear^13^, particularly when coastal waters near urban centres are often more exposed to anthropogenic pressures, and are therefore in greater need of protection^9^,^12^. Furthermore, the level of protection varies considerably between protected areas. For example, protection can range from no-take zones, to areas allowing different types and levels of activities such as tourism, fishing, petroleum and mineral extraction. Therefore, not all protected areas contribute equally to conservation objectives. While multiple use conservation zones provide some level of protection, extractive activities, by definition, have some level of impact. A recent review of 87 MPAs worldwide revealed that no-take areas (NTAs), when well enforced, long standing (>10 years), large (>100 km^2^), and isolated, provided the greatest benefits for biodiversity conservation^3^. However, it is currently estimated that NTAs cover less than 0.3% of the world oceans^14^.

The analyses presented herein explored how significant technical improvements in the conservation planning process can facilitate a more flexible and transparent conservation planning approach and deliver representative reserves while also balancing multiple stakeholder interests. The framework for systematic conservation planning has evolved significantly in the past 25 years as practitioners seek to formalise best practice. An initial framework described by Margules and Pressey^12^ focussed on key steps in the analysis and planning process while more recent frameworks have also integrated social, economic and political considerations^15^,^16^,^17^,^18^. There has been far greater uptake of planning outcomes when stakeholders are involved, particularly when they are involved early in the process^15^,^19^,^20^. Using a systematic conservation planning approach, we demonstrate technical improvements to support conservation planning using the marine protected area zoning system in north-west Australia as an illustrative case study. Australia has been a leader in marine conservation planning and management. For example, the Great Barrier Reef Marine Park (GBRMP) is internationally recognised as one of the best examples of the successful implementation of multiple use marine conservation planning^21^,^22^,^23^. Initially, zoning of the GBRMP protected areas concentrated in more remote sections of the park not suitable for trawling or other extractive uses^24^. The successful rezoning in 2004 demonstrated the value of a systematic conservation planning approach using robust data, conservation planning algorithms and extensive consultation with stakeholders^4^,^22^,^25^. In 2012, Australia’s National Representative System of Marine Protected Areas (NRSMPA) was released. Comprising more than a third of Australian marine waters, the NRSMPA created the world’s largest national coverage. However, there has been strong criticism that this network placed MPAs in remote areas that were of less importance to industry, affording little biodiversity protection for species and habitats most exposed to threatening processes^10^,^13^,^26^. Australia’s Exclusive Economic Zone (EEZ) is one of the largest in the world with a total marine area of around 10 million square kilometres. With limited ecological and biodiversity data to describe large sections of the marine area, it is perhaps not surprising that the justification for biodiversity protection is often lost in the face of well defined industry interests.

Australia’s north-west is characterised by a diverse range of marine habitats of high ecological value (e.g. mangrove forests, seagrass beds, coral reefs and sponge gardens)^27^,^28^. These environments support extremely diverse marine communities^29^,^30^ and provide important habitats for many vulnerable and threatened species including dugongs, turtles and whale-sharks^31^. However, owing to its remoteness, limited detailed, and spatially explicit, biodiversity data is available. The region is also one of the most economically significant marine area in Australia, producing the majority of the country’s oil and gas, with future projections indicating increasing output (Australian Gas Resource Assessment, 2012). Therefore, in Australia’s north-west, as in other marine regions of the world, identifying priority areas for conservation have been in direct conflict with the competing demands for natural resources. With global energy demands in 2035 predicted to increase by 37% (18% from oil and 53% from natural gas), regions that support large fossil fuel reserves, such as north-west Australia, will come under increasing pressure (Energy Outlook 2035). A global analysis examining regions at greatest risk from fossil fuel extraction has identified regions supporting high biodiversity and large fossil fuel reserves as having the greatest need of industry regulation, monitoring and conservation^32^. In addition, in August 2009, Australia experienced its largest petroleum industry oil spill within this region (approximately 4,750 tonnes) at the Montara wellhead platform drill rig owned by PTTEP Australasia (AMSA 2015). This spill was just eight months prior to the Deepwater Horizon oil spill (approximately 660,877 tonnes) in the Gulf of Mexico, the largest marine oil spill in the history of the offshore petroleum industry. Both events provided a timely reminder that environmental risk is also a critical consideration.

We undertook this research to demonstrate technical advances used to improve systematic conservation planning to facilitate uptake into conservation management processes. Improvements are detailed within five critical conservation planning steps. First, the best available biodiversity, socio-economic and environmental risk data was compiled through broad stakeholder consultation. Second, advanced spatial modelling techniques were applied to this data to derive comprehensive and spatially explicit biodiversity, socio-economic and environmental risk data layers. Third, quantitative conservation targets and design criteria appropriate for the region were set to demonstrate the approach. Fourth, a gap analysis was used to test whether the current proposed protected areas were ecologically representative. Finally, an improved systematic conservation planning tool (Marxan.net), engaging supercomputing resources to enable conservation scenarios with large datasets to be run in near-real time, was applied to the data. The overarching goal was to seek to achieve conservation targets with minimal impact to industry stakeholders.

## Methods

### Planning area

**Figure 1:**
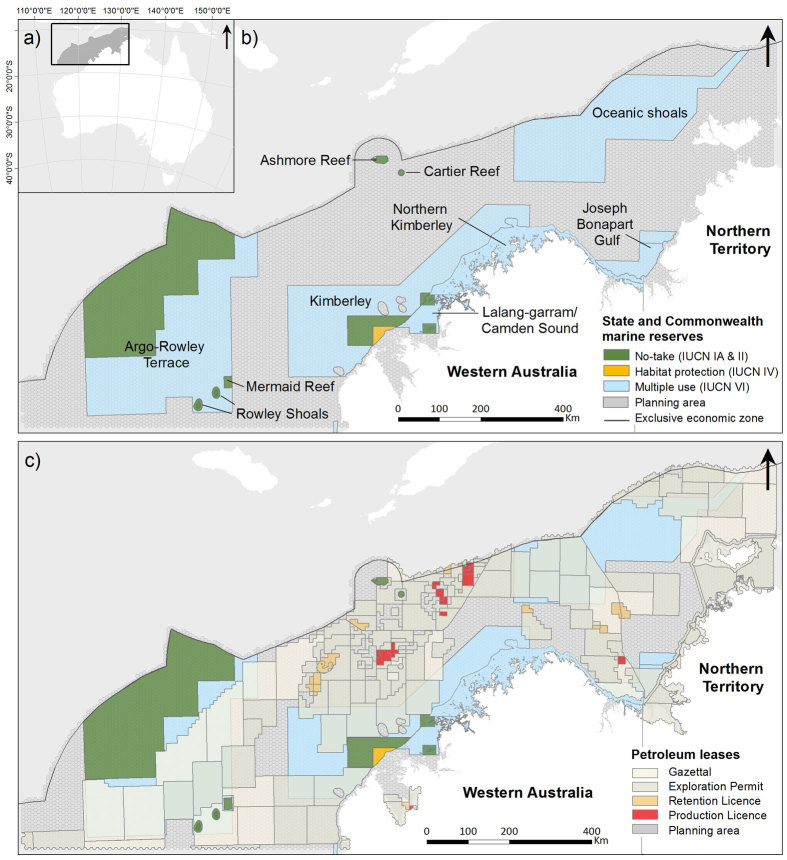
(**a**) Planning area. (**b**) Existing State and proposed Commonwealth marine reserves within the planning area. (**c**) Overlay of the petroleum leases and their current status within the region. (Figure created in ArcGIS 10.2 http://www.esri.com/).

The research illustrates technical advances in systematic marine conservation planning using the marine region of north-west Australia (Fig. 1). Proposed and existing no-take marine management zones currently cover 10.2% of this region. These include two state marine parks (Rowley Shoals and Lalang-garram/Camden Sound), three proposed state marine parks (Roebuck Bay, Horizontal Falls and Northern Kimberley), three Commonwealth marine reserves (Mermaid Reef, Ashmore Reef and Cartier Reef) and four proposed Commonwealth marine reserves (Argo-Rowley Terrace, Kimberley, Oceanic Shoals and Joseph Bonaparte Gulf). The size of the planning area (including state and commonwealth waters, but excluding areas of the Australian seabed beyond the EEZ) was 790,530 km^2^ divided into N = 12,162 planning units, each 65 km^2^ in size.

### Data collation

In total, 765 high resolution biodiversity data layers (including 674 species distributions and 91 environmental surrogates) were derived and used in the analysis (Table 1). Biodiversity data was collected through broad consultation with research, industry and government organisations. In total, 72,400 current and historical (dating back to 1982) species records with accurate locational information were collated. This included records from the Western Australian Museum, the Australian Institute of Marine Science (AIMS), PTTEP Australasia Ltd, The University of Western Australia, Curtin University, Department of Fisheries (Western Australia), INPEX Operations Australia Pty Ltd and Woodside Energy Limited. Species occurrence records represented 5 phyla, 14 classes, 41 orders and 119 families including fishes, corals, turtles, sea snakes, cetaceans, crustaceans, echinoderms and molluscs (Supplementary Table S1). Data were compiled from a number of survey techniques including diver based transects, baited underwater video systems, fisheries trap data and animal tracking data. Environmental data included the Integrated Marine and Coastal Regionalisation of Australia (IMCRA v4.0). IMCRA was developed as a framework for understanding Australia’s marine environment at a scale useful for regional planning. The regionalisation provides three levels of classification: provincial bioregions, mesoscale bioregions and geomorphic units. The three levels of the IMCRA classification were used as coarse filter surrogates for biological diversity. Best available bathymetry for the region was the General Bathymetric Chart of the Oceans, GEBCO_08 Grid, a global 30 arc-second grid. The bathymetry was divided into 11 ecologically meaningful depth categories based on expert opinion (0–10 m, 10–30 m, 30–60 m, 60–100 m, 100–200 m, 200–300 m, 300–500 m, 500–1000 m, 1000–2000 m, 2000–3000 m, 3000–6000 m). From the bathymetry a number of standard topographic measures (i.e. slope, aspect, curvature and surface area) known to be important in structuring and predicting species distributions were derived^33^,^34^,^35^. Continuous oceanographic measures were based on daily HYbrid Coordinate Ocean Model (HYCOM) 1/12° data collected from 2008 to 2013 (www.hycom.org). Tidal range data was obtained from the Oregon State University Tidal Inversion Software (OTIS using TPXO7.2 http://volkov.oce.orst.edu/tides/otis.html). The oceanographic layers were depth averaged and included temperature, velocity, salinity and tidal range.

**Figure 2:**
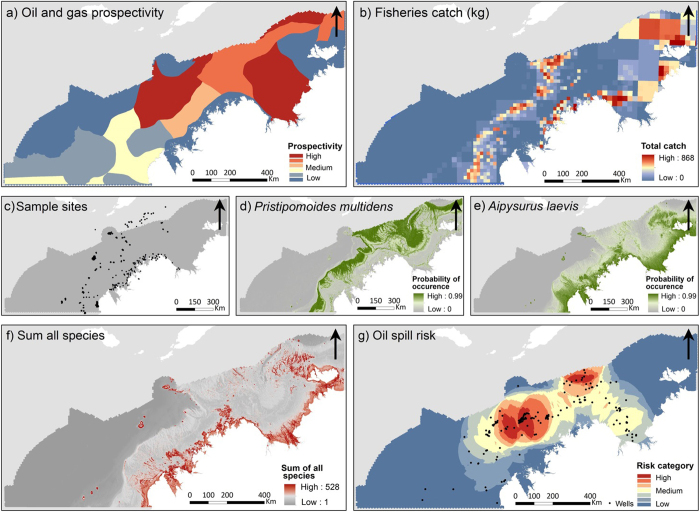
Spatial data sets. (**a**) Geoscience Australia (2008) relative petroleum prospectivity. (**b**) Commercial fisheries catch data (kg/km^2^). (**c**) Distribution of the occurence data collected across the region. (**d**) Predicted probability of occurence shown for *Pristopomoides multidens* (goldband jobfish). (**e**) Predicted probability of occurence shown for *Aipysus laevis* (olive seasnake). (**f**) Compiled predicted species data displaying sum of species occurrence across the region. (**g**) Modelled oil spill risk. (Figure created in ArcGIS 10.2 http://www.esri.com/).

Data available on the value of the petroleum resources within the region were limited. The layer used in the analysis was produced by Geoscience Australia and it provided broad scale relative prospectivity for the north and north-west of Australia (Fig. 2a). The data was based on quantitative basin evaluation work revised in 2009 by the Australian Government Department of the Environment and was provided with the caveat that the classification terms used represent a simplified qualitative assessment of petroleum prospectivity. Commercial fishery data were collected for the major fisheries operating within the region (pooled catch data in kg km^−2^ shown in Fig. 2b). A number of smaller fisheries exist in the region (e.g. trochus and mud crab). However, these could not be included in the analysis due to effort and catch data confidentiality issues. To capture spatial and temporal variability observed between years total catch data spanned a five year period (from 2007 to 2012).

### Spatial modelling

A total of 674 individual species distribution models were produced (Supplementary Table S1). As some historical museum data was presence-only, all the species data was modelled as presence-only data using maximum entropy modelling within the software, MaxEnt version 3.3.3^36^,^37^. MaxEnt has previously been used to model species distribution patterns across regions where data is sparse and where species accounts are presence-only records^34^. MaxEnt has consistently been found to provide predictive performance equal to that of the highest performing methods^38^. Presence only samples were modelled using the default settings with 10000 random background samples selected. The default settings are recommended when MaxEnt is used to model small or biased datasets as fine tuning can be unreliable^37^. Model performance was evaluated using the threshold-independent AUC (area under the curve) of the ROC (receiver operating characteristic) curve^39^ (Supplementary Table 1). An AUC value of 1.0 indicates perfect prediction while a value of 0.5 indicates the prediction is no better than chance alone^39^. Research into the minimum number of records required for accurate species distribution modelling has found minimum sample sizes as low as 3 for narrow-ranged species and 13 for widespread species appropriate^40^. However, this came with the caveat that the study area is ideal, balanced and orthogonal. Therefore, we chose a minimum sample of 20 occurrences to be conservative. Location of the occurrence data is detailed in Fig. 2c. AUC values indicated good predictive performance even though distributions included depth ranges and area outside that sampled. Example individual species distributions (i.e. for *Pristipomoides multidens* (Goldband snapper) AUC 0.916 and *Aipysurus laevis* (olive seasnake) AUC 0.935), are shown in Fig. 2d,e. A map showing the sum of all 674 predicted species occurrence data is shown in Fig. 2f. Environmental predictors used in the models included the bathymetry and topographic derivatives (Table 1). These variables where chosen as they provided high resolution data necessary to distinguish distributional patterns within geomorphological features and have been demonstrated to provide strong predictive performance for fish distributions^33^,^41^,^42^,^43^. Risk of exposure to an oil spill was included in the analysis with areas of high risk to be avoided where possible. We produced an oil spill risk model based on modelling a spill similar to that experienced at the Montara wellhead in 2009 (a 74 day spill with a 200 km trajectory) emanating from each of the existing wellheads in the region (Fig. 2g). The risk model was developed in ArcGIS 10.2 using the model builder to calculate cross tabulated areas of overlapping polygons.

### Setting conservation targets

Systematic conservation planning requires clear conservation targets. Specifically, how much of a species distribution or conservation feature will be protected within the network. The Convention on Biological Diversity (CBD) recommended a target of at least 10% of each of the world’s terrestrial and marine ecoregions. In a region where ecological processes are still poorly understood having sites set aside with the highest level of management and protection is a precautionary approach. Therefore the *a priori* focus of this assessment was to assess how the NTAs could be extended or reconfigured to meet 10% representation goals of all 765 species and environmental surrogates while minimising socio-economic costs and environmental risk. The planning process also requires decisions regarding design criteria. This refers to the spatial configuration of the network, including size, shape and number of areas. Design criteria, achieved through parameterisation of the Marxan algorithm, were used to meet conservation targets and balance costs while also achieving a compact and efficient marine reserve system^19^.

### Gap analysis

A gap analysis for the region was undertaken using available biophysical and socio-economic datasets^44^,^45^,^46^. Identification of gaps in conservation networks is dependent upon the accuracy of the biodiversity data and the ability of that data to indicate overall biodiversity. Ideally, analyses should be applied to the best available data and must explicitly incorporate uncertainty (i.e. predicted species distributions)^47^,^48^. Percent representation, within the current and proposed NTAs, was calculated for each species and environmental surrogate. For the gap analysis a representation target of 10% was chosen to indicate which species or environmental surrogates met CBD objectives^49^.

### Systematic conservation planning

We employed the new Marxan.net cloud infrastructure, a free online systematic conservation planning decision support tool^50^. Developed for Marxan^25^,^51^ the platform engages cloud technologies and supercomputing resources enabling conservation scenarios, with large datasets, to be run in near-real time. Marxan uses an optimisation algorithm to provide solutions that meet conservation objectives cost effectively. It achieves this using simulated annealing to select a set of potential conservation areas that meet a set of user-defined targets and costs efficiently. In this case it was used to meet our biodiversity targets of 10% protection, while minimising cost to industry and risk of oil spill. Cost was defined as the linear combination of opportunity losses to industry (i.e. fisheries and petroleum industries) and environmental risk. Cost layers were weighted by a cost multiplier to give equal weighting to the fishing and petroleum industries and environmental risk. The algorithm was parameterised following the protocol developed by Stewart and Possingham^19^. The protocol applies sensitivity analysis and calibration to parameterise Marxan to ensure conservation targets are met and near optimal solutions are found. Key parameters include; (1) the boundary length modifier (BLM), used to improve compactness of the reserves; (2) the species penalty factor (SPF), to ensure conservation targets are met; (3) planning unit cost (PU) and; (4) cost objective (CO). All parameters were balanced to achieve a compact and efficient marine reserve system.

### Conservation scenarios

Two systematic marine spatial planning scenarios were chosen to examine the adequacy of the NTAs and highlight any deficiencies in representation. Scenario 1 examined how the existing and proposed NTAs could be expanded to meet 10% conservation targets, while minimising socio-economic impacts and oil spill risk. Scenario 2 ignored the pre-existing NTAs and instead identified a new configuration of NTAs meeting 10% conservation targets, while also minimising socio-economic impacts and oil spill risk.

## Results

### Gap analysis

**Figure 3:**
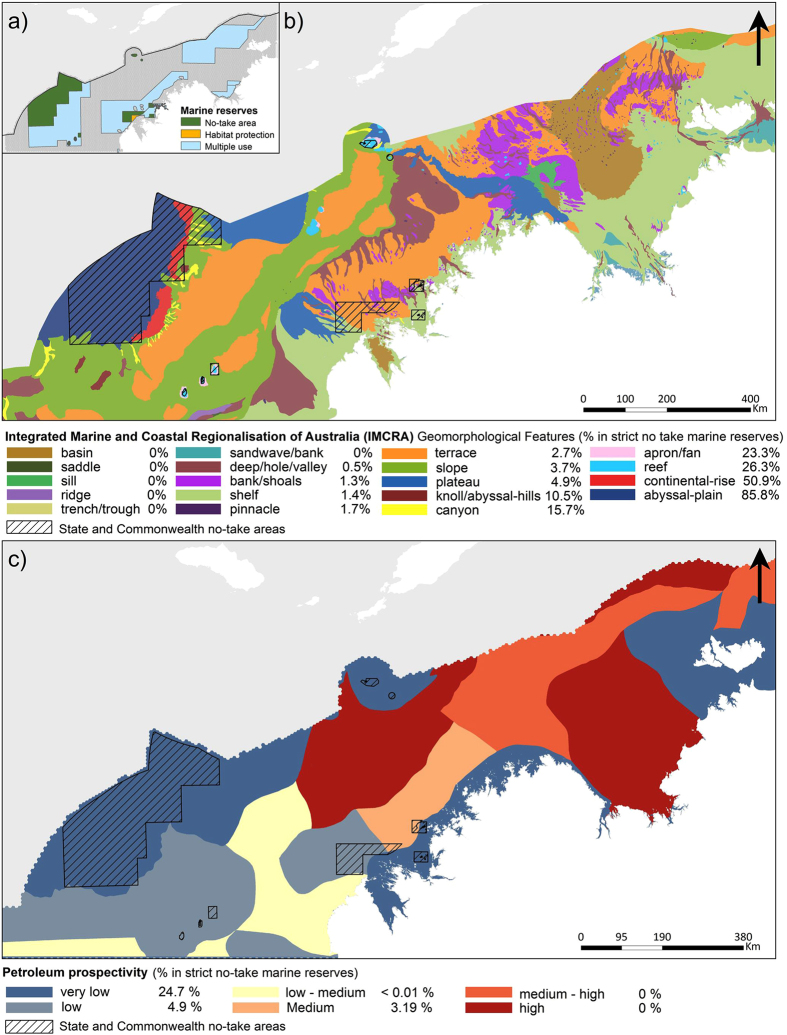
Gap analysis. (**a**) State and Commonwealth no take and multiple use marine reserves. (**b**) Gap analysis assessing representativeness within the no-take areas using IMCRA geomorphological features as surrogates for species diversity. (**c**) Gap analysis assessing impact on petroleum industry using the petroleum prospectively layer. (Figure created in ArcGIS 10.2 http://www.esri.com/).

The gap analysis revealed significant limitations in the representativeness of the existing (State and Commonwealth) and proposed (Commonwealth) no-take areas (NTAs) (Fig. 3). When examining the NTAs with respect to standard IMCRA geomorphological surrogates, 13 of the 19 biodiversity surrogates were either not included (e.g. basin, saddle, sill and ridge), or were severely under-represented (e.g. continental shelf 1.4%, banks and shoals 1.3% and pinnacle 1.7%; Fig. 3b). In contrast, some surrogates were considerably over-represented (i.e. abyssal-plain 85.8% and continental rise 50.9%). At the species level 98.2% of the 674 species included did not meet the 10% representation targets in the existing and proposed NTAs. In addition, more than a third of these (227 species) were very poorly represented with <2% coverage within the NTAs. Little overlap was observed between the current plan for NTAs and prospectivity for the petroleum industry (Fig. 3c).

### Conservation scenarios

**Figure 4:**
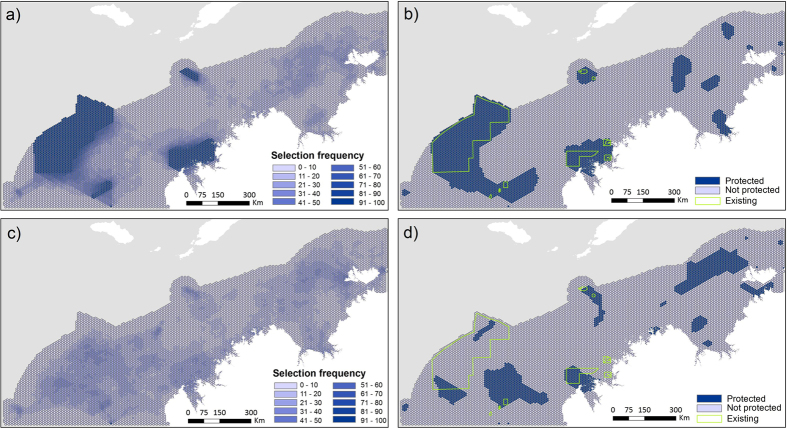
Conservation planning results using Marxan. (**A**) Scenario 1: planning unit selection frequency (scenario includes existing NTAs, cost layers and natural values). (**B**) Scenario 1: best solution. (**C**) Scenario 2: planning unit selection frequency (scenario includes cost layers and natural values). (**D**) Scenario 2: best solution. (Figure created in ArcGIS 10.2 http://www.esri.com/).

For scenario 1, existing and proposed NTAs were maintained and expanded to meet 10% targets while minimising industry costs and oil spill risk. Parameter testing found the optimal parametrisation to achieve a compact and efficient marine reserve system included; BLM = 6, SPF = 10, PU = area m^2^, CO = 100. For this scenario, the selection frequency of planning units concentrated within and around existing NTAs of the Agro-Rowley Terrace, Camden, Ashmore and Cartier reefs (Fig. 4a,b). This extension of the existing and proposed NTAs meets the minimum conservation target of 10% for all conservation features and would protect an area 2.5 times the proposed NTAs (185,510 km^2^).

Scenario 2 ignored the existing and proposed NTAs and re-examined where NTAs could be located to meet conservation targets while balancing industry costs and oil spill risk (Fig. 4c,d). Parameter testing found the optimal parametrisation included; BLM = 80, SPF = 1000, PU = area m^2^, CO = 10000. The best solution achieved the minimum conservation target of 10% for all conservation features, by protecting an area 1.6 times the current and proposed NTA (117,910 km^2^). Without the constraints of the existing and proposed NTAs, this alternative configuration would cover less of the Agro-Rowley Terrace, but more of the Oceanic Shoals (Fig 4d).

### Cost to industry

**Figure 5:**
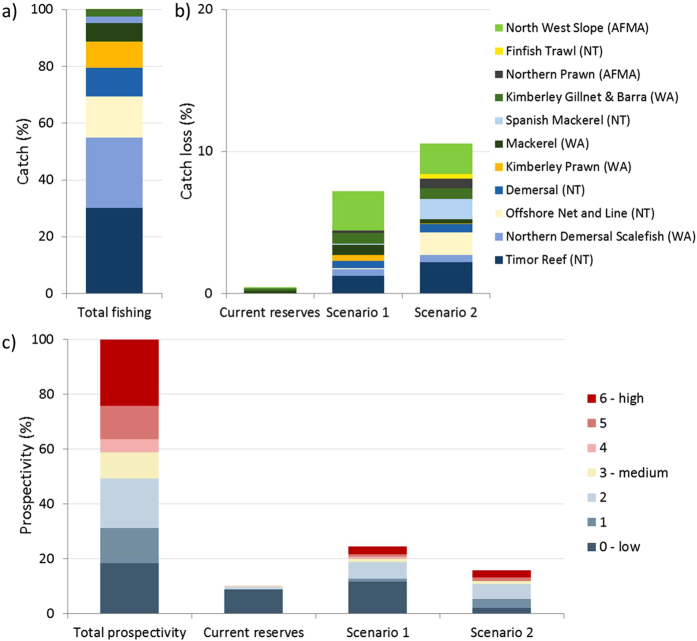
Predicted loss to industry calculated for the current no-take reserves and for the two reserve design scenarios. (**a**) Relative proportion of catch taken by each fishery. (**b**) Predicted catch loss (%) based on fisheries catch data (kg) collected over a five year period for each major fishery operating in the region. (**c**) Predicted loss of area prospective for oil and gas (%). (Figure created in ArcGIS 10.2 http://www.esri.com/).

The existing NTA configuration had very little impact (predicted loss of <0.4%) on existing commercial fisheries (Fig. 5a). In contrast, improving the representativeness of the natural values within the region would result in a potential loss to the industry of between 7.2% (scenario 1) and 10.5% (scenario 2) (Fig. 5b). Under scenario 1 and 2, it is the North West Slope fishery (managed by the Australian Fisheries Management Authority, AFMA) and the Timor Reef fishery (managed by Northern Territory Fisheries) that would be most affected. However, potential losses would be relatively small with estimated losses of up to 2.7% and 2.2% respectively. Data on prospective areas for oil and gas was at a very coarse resolution, but it was clear the existing NTA configuration has minimal costs to this industry (0.1% loss of area that is of medium to high prospectivity). Improving the representativeness of the natural values within the planning area would result in estimated industry losses of between 4.9% (scenario 2) and 5.6% (scenario 1) of area classified as medium to high prospectivity (Fig. 5c).

## Discussion

Conservation zones can be designed according to principles that ensure adequate representation across multiple features of conservation interest, while minimising adverse impacts to stakeholders. With the recent global trend of placing very large conservation zones in remote areas it is critical that these zones address primary threats and gaps in representation and not just minimise conflict^9^,^10^. While very large conservation zones in remote areas may meet CBD objectives in terms of areal extent, serious concerns have been raised as to whether they address primary threats and satisfy the fundamental goal of conservation: to ensure a fraction of all species and habitats are conserved^11^. The current network of NTAs representing Australia’s north-west marine region is no exception. The network meets CBD targets in terms of the percentage of area covered (10.2%). However, gap analysis revealed the network does not meet the CBDs recommended minimum target level of representation across all species distributions or features of conservation interest. To address this, we collated the best available biodiversity and socio-economic data, and applied advanced processing and modelling techniques to demonstrate how biodiversity values could be balanced with competing industry demands. We identified alternative NTA network configurations that better captured the region’s biodiversity while still minimising socio-economic costs. The approach allows for open and transparent discussions regarding the costs and benefits of conservation networks that meet CBD targets. It addresses primary threats and gaps in representation and facilitates near real-time negotiation with stakeholders to inform decision making.

Gaps in representation need to be assessed using the best available information. However, as for most remote marine regions, Australia’s north-west had very sparse species distribution data. In the absence of comprehensive species data, systematic conservation planning can be based on ‘coarse filter’ surrogates for biological diversity^52^,^53^. For example, broad scale geomorphological classifications and habitat maps may be used as surrogates with the assumption that adequate representation of each surrogate infers acceptable representation of biological diversity within the region^54^. This approach recognises that decisions on reserve design often need to be made without detailed species level knowledge. Robust species distribution modelling approaches are available that can handle sparse data and computing power, utilising parallel processing, can batch process large numbers of models. By compiling current and historical species distribution records across multiple institutions working within the region (i.e. research, government and industry) it was demonstrated how a comprehensive and accurate set of individual species distribution models could be derived. In total, the distribution of 674 species, representing 5 phyla, 14 classes, 41 orders and 119 families, were predicted. This species distribution modelling approach addresses one of the most critical limitations currently in conservation planning. It provides comprehensive, spatially explicit, individual species distribution data to ensure knowledge of the biological diversity represented by the region is adequate and defendable in the face of competing demands.

Defining explicit conservation targets and socio-economic objectives is essential for efficient systematic conservation planning. The distribution of NTAs was chosen using a representation target of 10% for each species’ distribution and biophysical surrogate. The success of a marine reserve in protecting biodiversity is dependent on a suite of factors both related to the individual species’ life history, the level of threat and the effectiveness of reserve management^3^. For our scenarios a 10% target was chosen to equate with the CBD goals of 10% representation. Data was collated, synthesised and targets set to perform a gap analysis which identified major gaps in representation of the current NTA network. For example, the majority of geomorphological surrogates used to represent regional biodiversity were either under-represented (<10%) or severely under-represented (<2%). Surrogates such as the continental shelf, pinnacles and banks and shoals are known to support unique and diverse biological communities but currently represent 1.4%, 1.7% and 1.3% of the NTAs, respectively^27^,^28^,^29^. The predictive species distribution modelling revealed that 98.2% of species were under-represented (<10%). In contrast, two surrogates were considerably over-represented (i.e. abyssal-plain 85.8% and continental rise 50.9%). In total, three quarters (75.3%) of the existing and proposed NTAs have been placed over a deep abyssal-plain and continental rise in depths of 3000 to 6000 metres. These habitats are remote and, due to their extreme depth, logistically and financially unattractive for petroleum or mineral extraction. Extractive technologies are enabling the offshore petroleum and mineral industries to operate at depths up to, and in excess of, 3000 metres. However, few industries in this region operate in depths greater than 200 metres. Therefore, the habitats and biodiversity most at risk are those exposed to anthropogenic activities on the continental shelf (0–200 metres). This bias in representation means that the habitats and biodiversity in greatest need of protection are currently offered the least.

With critical gaps in representation identified, the most important step was to identify improved solutions for conservation planning via transparent discussions regarding the costs and benefits of conservation networks that meet conservation targets. Two systematic marine conservation planning scenarios were examined in detail. Each highlighted factors to be carefully considered, and clearly demonstrated how improvements in comprehensiveness and representativeness would have distinct costs and benefits. Scenario 1 demonstrated that the pre-existing NTAs could be expanded to ensure all biodiversity values are represented with comparably low cost to industry. However, this approach was very inefficient. The NTAs would span a region at least 2.5 times that of the existing NTAs. Nonetheless one of the key factors of successful NTAs is longevity^55^; the longer a NTA has been established the more likely it will exhibit positive outcomes^2^,^3^,^56^,^57^. Hence conservation success might be achieved more rapidly by incorporating the existing and proposed NTAs within new networks. Alternatively, scenario 2 demonstrated how conservation objectives could be met more efficiently. By ignoring the location of current NTAs, the final NTA network in this scenario was more than 30% smaller. This scenario would also reduce ongoing costs required to maintain a larger network and enforce regulations. However, it would be more costly in terms of potential loss to industry and the cost of re-zoning the existing NTAs. Importantly, the systematic conservation planning approach adopted, allows different conservation scenarios to be rapidly quantified, facilitating open discussion with stakeholders.

In accordance with recommendations from the CBD, identifying priority areas for conservation needs to be balanced with socio-economic costs. The major industries operating within the region are the petroleum and commercial fishing industries. Therefore, the major socio-economic costs are opportunity losses to these industries. The potential costs to industry were itemised providing a platform for stakeholders to assess and discuss various outcomes. For example, scenario 1 and 2 were estimated to result in a total potential catch loss of 7.2% and 10.5% respectively, with the Timor Reef Fishery and the North West Slope fishery the two fisheries most affected by the proposed alternative networks of NTAs (potential losses up to 2.2% and 2.7% respectively). Questions that need to be addressed include whether these losses are acceptable in order to achieve conservation outcomes. To assist with these discussions, the analysis can be re-run blocking out critical areas identified for these fisheries, in order to establish whether alternative NTAs can be found that meet conservation targets and reduce the impact on these fisheries to acceptable levels.

Additional costs, considered less commonly in conservation planning, are the costs of anthropogenic and environmental impacts. In 2009, Australia experienced its largest petroleum industry oil spill within the north-west marine region providing an important reminder that risk is also a critical consideration for protected area planning. With risk of contamination treated as a cost, planning units less likely to be exposed will be preferentially selected. However, if those biodiversity features are restricted to areas considered high risk, they will be included in the reserved system to meet target representation. For example, recent research has revealed the exceptional diversity of the north-west oceanic banks and shoals, located within this region^29^,^58^. Research found these deeper (>30 m) coral reefs to support species richness 1.4 times that recorded for equivalent features on the Great Barrier Reef^58^. The species distribution modelling also revealed these deeper reefs as hotspots of marine biodiversity, with up to 505 of the 674 species predicted to occur on them. Currently, just 1.3% of the north-west oceanic banks and shoals occur within NTAs as opposed to approximately 29.3% of equivalent deeper reefs currently within NTAs on the Great Barrier Reef 59. Our analyses found both conservation scenarios addressed this gap in representation and included 10% of the oceanic banks and shoals. The most efficient reserve configuration (scenario 2) selected an area around Ashmore and Cartier Reefs thereby incorporating a number of banks and shoals. This area is probably the most contentious within the region. It is an area of high conservation value and high value to both the petroleum and fishing industries. In addition, from our oil spill risk modelling it is also a region with a high risk of exposure if there were another oil spill. Incorporating risk in a conservation planning analysis ensures the placement of NTAs will not only consider costs to industry, but will also consider environmental risk. The advantage being that future environmental risk scenarios have been explicitly considered and management actions, and in this case environmental emergency response plans, can be put in place to respond to these risks. Therefore, the results highlight a region that needs careful consideration and management to balance conservation values with industry activities.

Accuracy of the input data is a critical consideration. While we endeavoured to compile the most robust input data available at the time, we also identified improvements necessary for future management of the region. These include improving the predicted species distribution data and the accuracy and resolution of the cost data. The species distribution models were developed using relatively sparse data, therefore there is scope for improving the spatial, temporal and taxonomic extent of this information. Additional species distribution and environmental data have been, and are currently being collected for this region. However, this data has yet to be collated and made available for analyses of this kind^60^. For example, vast amounts of data exist in industry reports as part of mandatory environmental assessments. In addition, a number of large collaborative research programs are currently underway. Data collected from these programs could be fed directly into improving the species distribution models. In addition, with the availability of more data there may be scope to undertake a thorough climate change scenario analysis for the region examining past and future shifts in distributions. There is also an urgent need to improve the accuracy of the cost layers. The oil and gas prospectivity layer and some of the fisheries cost layers are provided at a very coarse resolution. Having coarse resolution data can disproportionally increase or decrease the cost of an area and bias the analysis.

To balance industry interests with marine conservation, environmental decision making must be founded on a solid conservation planning framework and good stakeholder engagement. The approach herein was underpinned by extensive data acquisition, data manipulation and systematic conservation planning to ensure protected areas address primary threats and gaps in representation, rather than merely meet a generic percentage-based areal target. This study was undertaken with active and ongoing engagement with key government and non-government research and industry partners. Analyses were used to highlight how excluding areas of high value to industry can result in a reserve system that is not representative. Technical advances to improve systematic conservation planning were used to analyse trade-offs and highlight opportunities to design representative, efficient and practical marine reserves that minimise potential loss to industry. By employing an advanced species distribution modelling approach, large amounts of sparse data can be used to provide a comprehensive set of spatially explicit species distributions to better represent a regions’ biodiversity. The research demonstrated how environmental risk can be incorporated into the analysis allowing for future risk scenarios to be fully considered. The use of a systematic conservation planning algorithm (Marxan) provided robust and independent decision support, delivering planning outcomes that efficiently achieved target levels of representation. In addition, the new Marxan.net accesses cloud technologies and parallel processing, in order for increasingly complex conservation scenarios, with large spatially explicit datasets, to be run rapidly and efficiently. This new development is critical as it enables a rigorous method to explore alternative planning scenarios in near-real time and facilitates open and transparent discussions regarding the costs and benefits of different conservation network scenarios. Advances such as these ensure we continue to progress conservation planning and explicitly balance industry interests and biodiversity conservation.

## Additional Information

**How to cite this article**: Moore, C. H. *et al*. Improving spatial prioritisation for remote marine regions: optimising biodiversity conservation and sustainable development trade-offs. *Sci. Rep.* **6**, 32029; doi: 10.1038/srep32029 (2016).

## Supplementary Material

Supplementary Information

## Figures and Tables

**Table 1:** Data used in Marxan analysis.

Type	Data	Description	Source or Algorithm
Species	Predicted occurrence	674 species distribution models	MaxEnt
Bioregional	Provincial bioregions	7 regions defined using biological, physical and spatial information.	IMCRA v4.0
	Mesoscale bioregions	13 regions defined using biological, physical and spatial information.	IMCRA v4.0
	Geomorphic units	19 areas that have similar geomorphological characteristics	IMCRA v4.0
Topographic	Bathymetry	General Bathymetric Chart of the Oceans, 30 arc-second grid	GEBCO_08 Grid 2010
	Slope	First derivative of elevation: average change in elevation/distance.	ArcGIS Arc/Info 10.2
	Aspect	Azimuthal direction of steepest slope	ArcGIS Arc/Info 10.2
	Curvature	Combined index of plan and profile curvature.	ArcGIS Arc/Info 10.2
	Plan curvature	Second derivative of elevation: concavity/convexity perpendicular to slope.	Jenness 2010
	Profile curvature	Second derivative of elevation: concavity/convexity parallel to slope	Jenness 2010
	Rugosity (surface area)	Surface area of the local neighbourhood and the ratio of the actual surface area to pixel area.	Jenness 2010
Oceanographic	Temperature	Depth averaged temperature 2008–2013	HYCOM
	Salinity	Depth averaged salinity 2008–2013	HYCOM
	Velocity	Depth averaged velocity 2008–2013	HYCOM
	Tidal range	Mean tidal range	TPX07–Atlas
Petroleum Industry	Prospectvity	Relative petroleum prospectivity of the north and north-west marine planning region	Geoscience Australia
Risk modelling	Oil spill risk	Modelled oil spill risk	ArcGIS Arc/Info 10.2
Fisheries	Northern Demersal Scalefish	Catch and effort data collected on a 10° and 60° grid	WA managed Fisheries
	Kimberley Gillnet and Barramundi	Catch and effort data collected on a 60° grid	WA managed Fisheries
	Mackerel	Catch and effort data collected on a 60° grid	WA managed Fisheries
	Kimberley Prawn	Catch and effort data collected on a 10° grid	WA managed Fisheries
	Spanish Mackerel	Catch and effort data collected on a 60° grid	NT managed fishery
	Offshore Net and Line	Catch and effort data collected on a 60° grid	NT managed fishery
	Demersal	Catch and effort data collected on a 60° grid	NT managed fishery
	Finfish Trawl	Catch and effort data collected on a 60° grid	NT managed fishery
	Timor Reef	Catch and effort data collected on a 60° grid	NT managed fishery
	North West Slope	Catch and effort data collected on a 60° grid	AFMA managed fishery
	Northern Prawn	Catch and effort data collected on a 60° grid	AFMA managed fishery
