# Supplementary material for: Improving spatial prioritisation for remote marine regions: optimising biodiversity conservation and sustainable development trade-offs
Source: Sci Rep. 2016 Aug 24;6:32029. doi: 10.1038/srep32029 (PMC4996080; doi:10.1038/srep32029)
Supplement: Supplementary Information [file srep32029-s1.pdf]

Improving spatial prioritisation for remote marine regions: optimising biodiversity conservation and sustainable development trade-offs.

Cordelia H. Moore*[1,2,3,4,5], Ben T. Radford[2,1], Hugh P. Possingham[6], Andrew J. Heyward[2,7], Romola R. Stewart[6], Matthew E. Watts[6], Jim Prescott[8], Stephen J. Newman[5], Euan S. Harvey[4], Rebecca Fisher[2,7], Clay W. Bryce[9], Ryan J. Lowe[1,10], Oliver Berry[3], Alexis Espinosa-Gayosso[7,11], Errol Sporer[5] and Thor Saunders[12]

[1]School of Earth and Environment and the UWA Oceans Institute, The University of Western Australia, 35 Stirling Highway, Crawley, WA 6009, Australia

[2]Australian Institute of Marine Science, UWA Oceans Institute (M096), 35 Stirling Highway, Crawley, Perth, WA 6009, Australia

[3]CSIRO Oceans and Atmosphere Flagship, PMB 5, Floreat, Western Australia, 6014, Australia

[4]Department of Environment and Agriculture, Curtin University, Bentley Campus, Perth, WA 6102, Australia

[5]Western Australian Fisheries and Marine Research Laboratories, Department of Fisheries, Government of Western Australia, P.O. Box 20, North Beach, WA, 6920, Australia

[6]ARC Centre of Excellence for Environmental Decisions, The University of Queensland, Brisbane, QLD 4072, Australia

[7]Oceans Institute, University of Western Australia, Crawley, WA 6009, Australia

[8]Australian Fisheries Management Authority, Darwin, NT 0801, Australia

[9]Western Australian Museum, Perth, WA 6986, Australia

[10]ARC Centre of Excellence for Coral Reef Studies, University of Western Australia, Crawley, Australia

[11]Civil, Environmental and Mining Engineering and the UWA Oceans Institute, University of Western Australia, Crawley, WA 6009, Australia.

[12]Department of Primary Industry and Fisheries, Darwin, NT 0801, Australia

Address for correspondence: Australian Institute of Marine Science, UWA Oceans Institute (M096), 35 Stirling Highway, Crawley, Perth, WA 6009, Australia, email cordelia.h.moore@curtin.edu.au

Supplementary Table 1: List of species included in the species distribution modelling. Table details species classification (Phylum, Class, Order, Family, Species), common name, testing AUC and number of records (n).

Phylum	Class	Order	Family	*Species*	Common name	Testing AUC	Records (n)
Arthropoda	Malacostraca	Decapoda	Diogenidae	*Calcinus gaimardii*	Hermit crab	0.9923	20
				Calcinus latens	Hidden hermit	0.9338	23
				Calcinus minutus	Hermit crab	0.952	30
				Dardanus lagopodes	Hermit crab	0.9802	27
Chordata	Actinopterygii	Anguilliformes	Labridae	*Halichoeres chrysus*	Golden wrasse	0.9957	22
				Halichoeres hortulanus	Checkerboard wrasse	0.9814	97
				Halichoeres marginatus	Dusky wrasse	0.9927	31
				Halichoeres melanurus	Hoevens wrasse	0.9805	47
				Halichoeres nebulosus	Cloud wrasse	0.9828	20
				Halichoeres nigrescens	Bubblefin wrasse	0.977	51
				Halichoeres prosopeion	Twotone wrasse	0.9798	110
				Halichoeres trimaculatus	Threespot wrasse	0.9888	24
				Halichoeres zeylonicus	Goldstripe wrasse	0.9954	58
			Muraenidae	*Gymnothorax favagineus*	Tessellate moray	0.9989	32
				Gymnothorax javanicus	Giant moray	0.8949	142
				Gymnothorax light sp Kimberley	Kimberley moray	0.8563	81
				Gymnothorax nudivomer	Yellowmouth moray	0.9095	104
				Gymnothorax sp Broome	Broome moray	0.8563	103
				Gymnothorax sp Kimberley	Kimberley moray	0.8563	184
			Synodontidae	*Saurida tumbil*	Common saury	0.8886	82
		Batrachoidiformes	Batrachoididae	*Halophryne diemensis*	Banded frogfish	0.997	20
		Beryciformes	Holocentridae	*Myripristis botche*	Blacktip soldierfish	0.8328	106
				Myripristis murdjan	Crimson soldierfish	0.9764	26
				Sargocentron caudimaculatum	Whitetail squirrelfish	0.4999	25
				Sargocentron rubrum	Red squirrelfish	0.9544	23
				Sargocentron spiniferum	Sabre squirrelfish	0.9957	33
		Gasterosteiformes	Fistulariidae	*Fistularia commersonii*		0.9846	32
		Perciformes	Acanthuridae	*Acanthurus auranticavus*	Orange-socket surgeonfish	0.8889	123
				Acanthurus blochii	Ringtail surgeonfish	0.9836	30
				Acanthurus dussumieri	Eyestripe surgeonfish	0.8694	110
				Acanthurus grammoptilus	Finelined surgeonfish	0.8686	331
				Acanthurus leucocheilus	Palelipped surgeonfish	0.9252	46
				Acanthurus mata	Elongate surgeonfish	0.8252	127
				Acanthurus nigricans	Whitecheek surgeonfish	0.9388	73
				Acanthurus olivaceus	Orangespot surgeonfish	0.9605	142
				Acanthurus pyroferus	Chocolate surgeonfish	0.9543	86
				Acanthurus thompsoni	Thompson's surgeonfish	0.9599	48
				Acanthurus xanthopterus	Yellowfis surgeonfish	0.9698	21
				Ctenochaetus striatus	Lined bristletooth	0.9739	189
				Naso annulatus	Ringtail unicornfish	0.9736	34
				Naso brachycentron	Humpback unicornfish	0.8606	61
				Naso brevirostris	Spotted unicornfish	0.9947	85
				Naso caesius	Silverblotched unicornfish	0.9976	46
				Naso hexacanthus	Sleek unicornfish	0.9769	135
				Naso lituratus	Clown unicornfish	0.9573	153
				Naso lopezi	Slender unicornfish	0.995	24
				Naso unicornis	Bluespine unicornfish	0.4588	29
				Naso vlamingii	Bignose unicornfish	0.9978	127
				Zebrasoma scopas	Brown tang	0.9857	85
				Zebrasoma veliferum	Sailfin tang	0.9925	36
			Apogonidae	*Apogon timorensis*	Timor cardinalfish	0.9995	23
				Cheilodipterus macrodon	Wolf cardinalfish	0.951	21
				Cheilodipterus quinquelineata	Five lined cardinalfish	0.8986	35
				Fowleria aurita	Ear cardinalfish	0.9923	37
			Blenniidae	*Aspidontus taeniatus*	Mimic blenny	0.7802	110
				Ecsenius bicolor	Bicolour combtooth blenny	0.9972	24
				Meiacanthus atrodorsalis	Eyelash fangblenny	0.9643	31

	Plagiotremus tapeinosoma	Piano fangblenny	0.9266	23
	Salarias fasciatus	Banded blenny	0.9685	21
Carangidae	*Alepes vari*	Herring scad	0.9615	44
	Atule mate	Yellowtail scad	0.9201	207
	Carangoides caeruleopinnatus	Onion trevally	0.8668	151
	Carangoides chrysophrys	Longnose trevally	0.851	276
	Carangoides ferdau	Blue trevally	0.9651	49
	Carangoides fulvoguttatus	Thicklip trevally	0.8121	302
	Carangoides gymnostethus	Bludger trevally	0.8793	420
	Carangoides hedlandensis	Bumpnose trevally	0.9873	53
	Carangoides malabaricus	Malabar trevally	0.6822	89
	Carangoides orthogrammus	Thicklip trevally1	0.9791	238
	Carangoides plagiotaenia	Barcheek trevally	0.9462	113
	Caranx heberi	Blacklip trevally	0.7792	103
	Caranx ignobilis	Giant trevally	0.8466	195
	Caranx lugubris	Black trevally	0.9559	35
	Caranx melampygus	Bluefin trevally	0.9714	241
	Caranx sexfasciatus	Bigeye trevally	0.7191	103
	Caranx tille	Tille trevally	0.7884	104
	Elagatis bipinnulata	Rainbow runner	0.9845	110
	Ganthanodon speciosus	Golden trevally	0.8807	299
	Gnathanodon speciosus	Golden trevally	0.945	61
	Scomberoides commersonnianus	Giant queenfish	0.9894	30
	Scomberomorus commerson	Spanish mackerel	0.9894	215
	Scomberomorus queenslandicus	School mackerel	0.9943	222
	Selaroides leptolepis	Yellowstripe scad	0.9947	109
	Seriola dumerili	Amberjack	0.899	306
	Seriola rivoliana	Highfin amberjack	0.9987	31
	Seriolina nigrofasciata	Balckbanded amberjack	0.9578	191
Chaetodontidae	*Chaetodon adiergastos*	Philippine butterflyfish	0.8694	49
	Chaetodon assarius	Western Butterflyfish	0.7742	103
	Chaetodon aureofasciatus	Goldstrip butterflyfish	0.8251	45
	Chaetodon auriga	Threadfin butterflyfish	0.8251	95
	Chaetodon baronessa	Triangular butterflyfish	0.9681	35
	Chaetodon bennetti	Eclipse butterflyfish	0.9463	33
	Chaetodon ephippium	Saddle butterflyfish	0.9538	47
	Chaetodon kleinii	Kleins butterflyfish	0.976	218
	Chaetodon lineolatus	Lined butterflyfish	0.9544	86
	Chaetodon lunula	Racoon butterflyfish	0.9544	80
	Chaetodon lunulatus	Pinstripe butterflyfish	0.9544	82
	Chaetodon meyeri	Meyers butterflyfish	0.9533	34
	Chaetodon ornatissimus	Ornate butterflyfish	0.9775	80
	Chaetodon oxycephalus	Spotnape butterflyfish	0.8549	21
	Chaetodon punctatofasciatus	Spotbanded butterflyfish	0.9446	29
	Chaetodon semeion	Dotted butterflyfish	0.7886	24
	Chaetodon sp Kimberly	Kimberley butterflyfish	0.9161	81
	Chaetodon speculum	Ovalspot butterflyfish	0.9244	26
	Chaetodon trifascialis	Chevron butterflyfish	0.9351	113
	Chaetodon ulietensis	Doublesaddle butterflyfish	0.9262	93
	Chaetodon unimaculatus	Teardrop butterflyfish	0.967	23
	Chaetodon vagabundus	Vagabond butterflyfish	0.9278	25
	Chelmon marginalis	Margined coralfish	0.951	133
	Coradion altivelis	Highfin coralfish	0.4485	92
	Coradion chrysozonus	Orangebanded coralfish	0.7983	120
	Forcipiger flavissimus	Forceps fish	0.9939	66
	Forcipiger longirostris	Longnose butterflyfish	0.9848	46
	Hemitaurichthys polylepis	Pyramid butterflyfish	0.9992	27
	Heniochus acuminatus	Longfin bannerfish	0.96	250
	Heniochus chrysostomus	Pennant bannerfish	0.9807	29
	Heniochus singularius	Singlar bannerfish	0.9983	35
	Heniochus varius	Horned bannerfish	0.9828	43
	Parachaetodon ocellatus	Ocellate butterflyfish	0.8035	175
Cirrhitidae	*Paracirrhites forsteri*	Freckled hawkfish	0.9987	45
Clupeidae	*Anodontostoma chacunda*	Gizzard Shad	0.7065	86
Echeneidae	*Echeneis naucrates*	Sharksucker	0.9513	369
Ephippidae	*Platax batavianus*		0.9493	202
	Platax teira	Roundface batfish	0.6166	106

Gerreidae	*Gerres filamentosus*	Threadfin silverbiddy	0.96	81
Glaucosomatidae	*Glaucosoma buergeri*	Norther pearl perch	0.9161	189
Gobiidae	*Amblygobius bynoensis*	Bynoe goby	0.9597	28
	Eviota queenslandica	Queensland evoita	0.9959	22
	Istigobius ornatus	Ornate sand goby	0.9924	21
Haemulidae	*Diagramma pictum*	Painted sweetlips	0.8741	430
	Plectorhinchus chaetodonoides	Spotted sweetlips	0.9852	27
	Plectorhinchus gibbosus	Brown sweetlips	0.921	191
	Plectorhinchus polytaenia	Ribbon sweetlips	0.997	88
Labridae	*Bodianus axillaris*	Coral pigfish	0.9846	42
	Bodianus diana	Dianas pigfish	0.9858	41
	Bodianus mesothorax	Eclipse pigfish	0.8771	20
	Bodianus perditio	Goldspot pigfish	0.8667	305
	Cetoscarus bicolor	Bicolour parrotfish	0.8848	37
	Cheilinus chlorourus	Floral maori wrasse	0.9202	33
	Cheilinus fasciatus	Redbreast maori wrasse	0.9062	197
	Cheilinus trilobatus	Tripletail maori wrasse	0.9667	60
	Cheilinus undulatus	Humheaded maori wrasse	0.942	39
	Chlorurus bleekeri	Bleekers parrotfish	0.9758	39
	Chlorurus sordidus	Greenfin parrotfish	0.9528	79
	Choerodon cauteroma	Bluespotted tuskfish	0.8579	108
	Choerodon cephalotes	Purple tuskfish	0.9308	32
	Choerodon cyanodus	Blue tuskfish	0.9603	71
	Choerodon jordani	Dagger tuskfish	0.8717	57
	Choerodon schoenleinii	Blackspot tuskfish	0.8199	118
	Choerodon venustus	Venus tuskfish	0.9826	81
	Choerodon zamboangae	Eyebrow tuskfish	0.7789	103
	Cirrhilabrus exquisitus	Exquisite wrasse	0.9343	27
	Cirrhilabrus randalli	Randalls wrasse	0.9311	48
	Cirrhilabrus sp1		0.9637	107
	Cirrhilabrus sp2		0.9081	33
	Conniella apterygia	Connies wrasse	0.864	82
	Coris aygula	Redblotched wrasse	0.9814	20
	Coris gaimard	Clown wrasse	0.962	58
	Dotalabrus aurantiacus	Castelnaus wrasse	0.967	81
	Epibulus insidiator	Slingjaw wrasse	0.8464	47
	Gomphosus varius	Birdnose wrasse	0.9895	54
	Hemigymnus fasciatus	Fiveband wrasse	0.9959	33
	Hemigymnus melapterus	Thicklip wrasse	0.9819	52
	Hologymnosus doliatus	Pastel slender wrasse	0.4931	50
	Iniistius pavo	Blue razorfish	0.9981	20
	Labrichthys unilineatus	Oneline wrasse	0.9256	21
	Labroides bicolor	Bicolor cleanerfish	0.9952	59
	Labroides dimidiatus	Common cleanerfish	0.9542	485
	Labropsis xanthonota	Yellowback tubelip	0.4924	20
	Leptojulis cyanopleura	Shoulderspot wrasse	0.8869	101
	Novaculichthys taeniourus	Carpet wrasse	0.9625	20
	Oxycheilinus celebicus	Slender maori wrasse	0.968	176
	Oxycheilinus digrammus	Violetine maori wrasse	0.9523	124
	Oxycheilinus Stripey banded	Maori wrasse sp	0.9857	46
	Oxycheilinus unifasciatus	Ringtail maori wrasse	0.9865	61
	Pseudocheilinus evanidus	Pinstrip wrasse	0.9565	31
	Pseudocheilinus hexataenia	Sixline wrasse	0.9803	40
	Pseudodax moluccanus	Chiseltooth wrasse	0.6999	52
	Pseudojuloides severnsi		0.9987	52
	Scarus flavipectoralis	Yellowfin parrotfish	0.9753	131
	Scarus forsteni	Whitespot parrotfish	0.7	57
	Scarus ghobban	Bluebarred parrotfish	0.9296	230
	Scarus oviceps	Darkcap parrotfish	0.9933	53
	Scarus rivulatus	Surf parrotfish	0.5617	24
	Scarus rubroviolaceus	Blackvein parrotfish	0.992	48
	Scarus schlegeli	Schlegels parrotfish	0.9224	250
	Scarus sp Broome	Broome parrotfish sp	0.9656	103
	Scarus sp2 Broome	Broome parrotfish sp2	0.9153	103
	Suezichthys cyanolaemus	Bluethroat rainbow wrasse	0.975	81
	Thalassoma blycephalum	Ladder wrasse	0.9908	33
	Thalassoma hardwicke	Sixbar wrasse	0.9978	43
	Thalassoma jansenii	Jansens wrasse	0.9643	25
	Thalassoma lunare	Moon wrasse	0.9575	100

Leiognathidae	*Leiognathus longispinis*	Longspine ponyfish	0.989	40
Lethrinidae	*Gymnocranius elongatus*	Swallowtail seabream	0.9706	107
	Gymnocranius grandoculis	Robinsons seabream	0.9316	486
	Lethrinus amboinensis	Ambon emperor	0.966	334
	Lethrinus atkinsoni	Yellowtail emperor	0.9625	188
	Lethrinus bitaeniatus		0.945	103
	Lethrinus erythracanthus	Orangespotted emperor	0.9643	148
	Lethrinus erythropterus	Longfin emperor	0.9643	62
	Lethrinus genivittatus	Threadfin emperor	0.9952	83
	Lethrinus laticaudis	Grass emperor	0.9542	21
	Lethrinus lentjan	Redsnout emperor	0.9358	397
	Lethrinus microdon	Smalltooth emperor	0.9686	24
	Lethrinus miniatus	Sweetlip emperor	0.9302	84
	Lethrinus nebulosus	Spangled emperor	0.923	366
	Lethrinus obsoletus	Orangestriped emperor	0.9987	24
	Lethrinus olivaceus	Longnose emperor	0.9151	796
	Lethrinus punctulatus	Pink ear emperor	0.9015	138
	Lethrinus ravus	Drab emperor	0.9392	462
	Lethrinus rubrioperculatus	Spotcheek emperor	0.9629	733
	Lethrinus semicinctus	Blackblotch emperor	0.987	378
	Lethrinus sp 3	Emperor sp 3	0.9031	115
	Lethrinus sp Kimberley	Kimberley emperor	0.9173	81
	Lethrinus sp Kimberley darkblotch	Kimberley darkblotch emperor	0.9968	81
	Monotaxis grandoculis	Bigeye seabream	0.9633	128
	Wattsia mossambica	Mozambique seabream	0.9642	333
Lutjanidae	*Aphareus rutilans*	Rusty jobfish	0.8282	148
	Aprion virescens	Green jobfish	0.9691	488
	Caesio cuning	Yellowtail fusilier	0.8184	164
	Caesio teres	Blue fusilier	0.9488	52
	Lipocheilus carnolabrum	Tang snapper	0.8962	103
	Lutjanus argentimaculatus	Mangrove Jack	0.9099	98
	Lutjanus bitaeniatus	Indonesian snapper	0.9215	306
	Lutjanus bohar	Red bass	0.9917	389
	Lutjanus carponotatus	Stripey snapper	0.9523	176
	Lutjanus decussatus	Checkered snapper	0.9685	154
	Lutjanus erythropterus	Crimson snapper	0.8886	312
	Lutjanus gibbus	Paddletail	0.9897	174
	Lutjanus kasmira	Bluestriped snapper	0.4882	28
	Lutjanus lemniscatus	Darktail snapper	0.8881	338
	Lutjanus malabaricus	Saddletail snapper	0.8742	393
	Lutjanus quinquelineatus	Fiveline snapper	0.9266	120
	Lutjanus rivulatus	Maori snapper	0.9367	288
	Lutjanus russelli	Moses snapper	0.9212	409
	Lutjanus sebae	Red emperor	0.9059	428
	Lutjanus sp kimberley	Kimberley sp	0.9982	81
	Lutjanus vitta	Brownstripe snapper	0.9225	438
	Macolor macularis	Midnight snapper	0.9709	107
	Macolor niger	Black and white snapper	0.9968	66
	Pristipomoides filamentosus	Rosy snapper	0.9198	242
	Pristipomoides multidens	Goldband snapper	0.9164	402
	Pristipomoides typus	Sharptooth snapper	0.8903	329
	Pterocaesio chrysozona	Yellowband fusilier	0.9917	82
	Pterocaesio digramma	Doublelined fusilier	0.9908	24
	Pterocaesio marri	Bigtail fusilier	0.5	25
	Pterocaesio sp Broome	Broome fusilier sp	0.9309	103
	Pterocaesio tile	Neon fusilier	0.6756	29
	Pterocaesio trilineata	Threestripe fusilier	0.7371	29
	Symphorus nematophorus	Chinamanfish	0.9025	230
Malacanthidae	*Hoplolatilus cuniculus*	Green tilefish	0.5	23
	Malacanthus brevirostris	Flagtail blanquillo	0.9006	62
	Malacanthus latovittatus	Blue blanquillo	0.9988	37
Microdesmidae	*Nemateleotris magnifica*	Red firegoby	0.4999	20
	Ptereleotris evides	Arrow dartgoby	0.9836	30
	Ptereleotris sp Kimberley	Kimberley dartgoby sp	0.9215	81
Mullidae	*Parupeneus barberinoides*	Bicolour goatfish	0.9859	249
	Parupeneus barberinus	Dot and dash goatfish	0.9859	22
	Parupeneus chrysopleuron	Rosy goatfish	0.9358	81
	Parupeneus cyclostomus	Goldsaddle goatfish	0.968	136
	Parupeneus heptacanthus	Opalescent goatfish	0.9629	241
	Parupeneus indicus	Yellowspot goatfish	0.9156	208

	Parupeneus multifasciatus	Banded goatfish	0.9923	491
	Parupeneus pleurostigma	Sidespot goatfish	0.9911	73
	Upeneus tragula	Bartail goatfish	0.8989	25
Nemipteridae	*Nemipterus furcosus*	Rosy threadfin bream	0.8848	217
	Nemipterus hexodon	Ornate threadfin bream	0.9532	24
	Pentapodus emeryii	Purple threadfin bream	0.983	340
	Pentapodus nagasakiensis	Japanese threadfin bream	0.9657	210
	Pentapodus paradiseus	Paradise threadfin bream	0.923	82
	Pentapodus porosus	Northwest threadfin bream	0.9806	206
	Pentapodus vitta	Western butterfish	0.9842	36
	Scaevius milii	Coral monocle bream	0.9692	28
	Scolopsis bilineata	Two line monocle bream	0.975	174
	Scolopsis margaritifer	Pearly monocle bream	0.8197	102
	Scolopsis monogramma	Rainbow monocle bream	0.815	120
	Scolopsis xenochrous	Oblique bar monocle bream	0.9478	229
Pinguipedidae	*Parapercis clathrata*	Spothead grubfish	0.9755	20
	Parapercis multiplicata	Doublestitch grubfish	0.9923	42
	Parapercis nebulosa	Pinkbanded grubfish	0.9706	215
	Parapercis sp Broome	Broome parapercis	0.9463	103
	Parapercis sp1	Parapercis sp1	0.99	61
Pomacanthidae	*Apolemichthys trimaculatus*	Threespot angelfish	0.9017	301
	Centropyge bicolor	Bicolour angelfish	0.9417	154
	Centropyge bispinosa	Coral beauty	0.9977	29
	Centropyge nox	Midnight angelfish	0.8153	23
	Centropyge tibicen	Keyhole angelfish	0.963	53
	Centropyge vroliki	Pearl scaled angelfish	0.9484	61
	Chaetodontoplus duboulayi	Scribbled angelfish	0.8666	123
	Chaetodontoplus mesoleucus	Vermiculate angelfish	0.9364	23
	Chaetodontoplus personifer	Yellowtail angelfish	0.763	184
	Genicanthus lamarck	Lamarcks anglefish	0.9828	62
	Pomacanthus imperator	Emperor angelfish	0.9587	363
	Pomacanthus sexstriatus	Sixband angelfish	0.9284	153
	Pomacentrus adelus	Obscure damsel	0.9732	29
	Pomacentrus alexanderae	Alexanders damsel	0.8962	93
	Pomacentrus bankanensis	Speckled damsel	0.9885	40
	Pomacentrus coelestis	Neon damsel	0.985	187
	Pomacentrus lepidogenys	Scaley damsel	0.9982	24
	Pomacentrus littoralis	Smoky damsel	0.4992	20
	Pomacentrus milleri	Millers damsel	0.9886	46
	Pomacentrus moluccensis	Lemon damsel	0.9939	37
	Pomacentrus nagasakiensis	Blue scribbled damsel	0.9392	105
	Pomacentrus nigromanus	Goldback damsel	0.9883	24
	Pomacentrus nigromarginatus	Goldback damsel	0.9883	25
	Pomacentrus philippinus	Philippine damsel	0.9964	35
	Pomacentrus vaiuli	Princess damsel	0.9922	61
	Pygoplites diacanthus	Regal Angelfish	0.9565	119
Pomacentridae	*Abudefduf bengalensis*	Bengal sergeant	0.9673	57
	Acanthochromis polyacanthus	Spiny chromis	0.9726	74
	Amblyglyphidodon aureus	Golden damsel	0.9766	24
	Amblyglyphidodon leucogaster	Golden damsel	0.9545	61
	Amphiprion clarkii	Clarks anemonefish	0.924	26
	Chromis atripes	Darkfin puller	0.8806	24
	Chromis cinerascens	Green puller	0.9333	20
	Chromis fumea	Smoky puller	0.7386	111
	Chromis margaritifer	Whitetail puller	0.9278	63
	Chromis ternatensis	Swallowtail puller	0.9661	43
	Chromis viridis	Blue green puller	0.9739	25
	Chromis weberi	Webers puller	0.964	79
	Chromis westaustralis	West Australian puller	0.9956	88
	Chromis xanthura	Pale tail puller	0.9089	54
	Chrysiptera cyanea	Blue demoiselle	0.9556	24
	Chrysiptera hemicyanea	Azure demoiselle	0.9207	29
	Chrysiptera rex	Pink demoiselle	0.8882	23
	Dascyllus aruanus	Banded humbug	0.9976	33
	Dascyllus reticulatus	Headband humbug	0.9956	59

		Dascyllus trimaculatus	Threespot humbug	0.9721	58
		Dischistodus prosopotaenia	Honeyhead damsel	0.9954	23
		Neoglyphidodon melas	Black damsel	0.9495	64
		Neopomacentrus azysron	Yellowtail demoiselle	0.9944	34
		Neopomacentrus filamentosus	Brown demoiselle	0.9798	22
		Plectroglyphidodon dickii	Dick's damsel	0.9968	21
		Plectroglyphidodon johnstonianus	Johnston damsel	0.6536	21
		Plectroglyphidodon lacrymatus	Jewel damsel	0.9993	21
		Stegastes nigricans	Dusty gregory	0.4882	21
		Stegastes obreptus	Western gregory	0.931	23
	Pseudochromidae	*Congrogadus subducens*	Carpet eel blenny	0.9316	24
		Pseudochromis fuscus	Dusky dottyback	0.9941	34
		Pseudochromis paccagnellae	Royal dottyback	0.9864	34
	Rachycentridae	*Rachycentron canadum*	Cobia	0.943	299
	Scombridae	*Gymnosarda unicolor*	Dogtooth tuna	0.9674	103
	Serranidae	*Aethaloperca rogaa*	Redmouth grouper	0.9626	111
		Cephalopholis argus	Peacock rockcod	0.9452	55
		Cephalopholis boenack	Brownbarred rockcod	0.9907	33
		Cephalopholis cyanostigma	Blue spotted rockcod	0.9714	25
		Cephalopholis leopardus	Leopard rockcod	0.9201	125
		Cephalopholis miniata	Coral rockcod	0.9237	49
		Cephalopholis sonnerati	Tomoato rockcod	0.9045	226
		Cephalopholis urodeta	Flagtail rockcod	0.9753	94
		Cromileptes altivelis	Barramundi cod	0.8668	222
		Diploprion bifasciatum	Barred soapfish	0.9798	82
		Epinephelus amblycephalus	Banded grouper	0.9359	304
		Epinephelus areolatus	Yellowspotted rockcod	0.947	310
		Epinephelus bilobatus	Frostback rockcod	0.901	424
		Epinephelus bleekeri	Duskytail grouper	0.9234	304
		Epinephelus chlorostigma	Brownspotted grouper	0.9839	46
		Epinephelus coioides	Goldspotted rockcod	0.8653	404
		Epinephelus fasciatus	Blacktip rockcod	0.9844	42
		Epinephelus maculatus	Highfin grouper	0.9876	23
		Epinephelus malabaricus	Blackspotted rockcod	0.9114	316
		Epinephelus merra	Birdwire rockcod	0.9966	34
		Epinephelus morrhua	Comet grouper	0.9713	220
		Epinephelus multinotatus	Rankin cod	0.8886	388
		Epinephelus polyphekadion	Camouflage grouper	0.8233	241
		Epinephelus rivulatus	Chinaman rockcod	0.9338	108
		Epinephelus sp Broome	Broome Rockcod	0.9467	103
		Epinephelus stictus	Blackdotted grouper	0.946	103
		Epinephelus tukula	Potato rockcod	0.8702	393
		Gracila albomarginata	Thinspine grouper	0.9983	20
		Plectropomus areolatus	Spotted cod	0.9897	28
		Plectropomus laevis	Bluespotted coral trout	0.9806	29
		Plectropomus leopardus	Coral trout	0.9437	410
		Plectropomus maculatus	Barcheek coral trout	0.8582	319
		Plectropomus oligacanthus	Vermicular cod	0.98	95
		Pseudanthias sp Broome	Broome basslet sp	0.9745	103
		Pseudanthias sp2 Broome	Broome basslet sp2	0.9693	103
		Pseudanthias tuka	Purple queen	0.9972	28
		Variola albimarginata	White edge coronation trout	0.9776	299
		Variola louti	Yellow edge coronation trout	0.9878	94
	Siganidae	*Siganus argenteus*	Forktail rabbitfish	0.9547	126
		Siganus doliatus	bluelined rabbitfish	0.8222	42
		Siganus puellus	Masked rabbitfish	0.9713	26
		Siganus punctatissimus	Finespotted rabbitfish	0.9963	21
		Siganus punctatus	Spotted rabbitfish	0.9963	48
		Siganus vulpinus	Foxface	0.9802	35
	Sparidae	*Argyrops spinifer*	Frypan Bream	0.796	190
	Sphyraenidae	*Sphyraena barracuda*	Akerstroms sea pike	0.9352	56
		Sphyraena sp Broome	Broome pike sp	0.9793	103
	Zanclidae	*Zanclus cornutus*	Moorish idol	0.9548	328
Siluriformes	Plotosidae	*Paraplotosus albilabris*	Sailfin catfish	0.9643	20
Tetraodontiformes	Balistidae	*Abalistes stellaris*	Starry triggerfish	0.8668	201

				Abalistes stellatus		0.8668	327
				Balistapus undulatus	Orangestripe triggerfish	0.9637	239
				Balistoides conspicillum	Clown triggerfish	0.9771	89
				Balistoides viridescens	Titan triggerfish	0.95	215
				Melichthys vidua	Pinktail triggerfish	0.9926	34
				Odonus niger	Redtooth triggerfish	0.9923	84
				Pseudobalistes flavimarginatus	Yellowmargin triggerfish	0.9943	43
				Pseudobalistes fuscus	Yellowspot triggerfish	0.9941	50
				Sufflamen bursa	Pallid triggerfish	0.9266	65
				Sufflamen chrysopterum	Eye stripe triggerfish	0.9367	120
				Sufflamen fraenatum	Bridled triggerfish	0.9668	474
			Monacanthidae	*Aluterus monoceros*	Unicorn leatherjacket	0.9875	87
				Aluterus scriptus	Scribbled leatherjacket	0.9188	161
				Cantherhines dumerilii	Barred leatherjacket	0.9732	27
				Paramonacanthus otisensis	Dusky leatherjacket	0.9878	20
			Tetraodontidae	*Arothron hispidus*	Stars and stripes puffer	0.8825	23
				Arothron nigropunclatus		0.9846	20
				Lagocephalus lunaris	Rough golden toadfish	0.9302	387
				Lagocephalus sceleratus	Silver toadfish	0.9173	197
	Chondrichthyes	Carcharhiniformes	Carcharhinidae	*Carcharhinus albimarginatus*	Silvertip shark	0.7079	253
				Carcharhinus amblyrhynchos	Grey reef shark	0.8744	351
				Carcharhinus limbatus	Blacktip shark	0.7873	105
				Carcharhinus plumbeus	Sandbar shark	0.7612	186
				Carcharhinus sp Broome	Broome shark sp	0.9943	103
				Carcharhinus sp kimberley	Kimberley shark sp	0.9716	81
				Carcharhinus tilstoni	Australian blacktip shark	0.7884	33
				Galeocerdo cuvier	Tiger shark	0.8352	192
				Triaenodon obesus	Whiptip reefshark	0.9319	374
			Sphyrnidae	*Sphyrna lewini*	Scalloped hammerhead	0.915	103
				Sphyrna mokarran	Great hammerhead	0.9669	28
			Triakidae	*Hemitriakis sp Broome*	Broome shark	0.8807	103
		Myliobatiformes	Dasyatidae	*Dasyatis kuhlii*	Bluespotted maskray	0.9635	85
				Taeniura lymma	Bluespotted fantail ray	0.9212	54
				Taeniura meyeni	Blotched fantail ray	0.8683	206
		Orectolobiformes	Ginglymostomatidae	*Nebrius ferrugineus*	Tawny shark	0.9123	247
			Stegostomatidae	*Stegostoma fasciatum*	Zebra shark	0.9779	92
		Rhynobatiformes	Rynchobatidae	*Rhynchobatus djiddensis*	Whitespotted wedgefish	0.8413	207
	Gnathostomata	Chondrichthyes	Odontaspididae	*odontaspis ferox*	Sandtiger shark	0.945	81
	Mammalia	Cetacea	Balaenopteridae	*Megaptera novaeangliae*	Humpback whale	0.5	1565
			Delphinidae	*Stenella longirostris*	Spinner dolphin	0.8881	48
				Tursiops aduncus	Long beaked bottle nosed dolphin	0.998	60
	Reptilia	Squamata	Hydrophiidae	*Aipysurus laevis*	Olive seasnake	0.9354	210
				seasnake banded	Banded seasnake	0.9897	104
		Testudines	Cheloniidae	*Chelonia mydas*	Green turtle	0.945	7860
				Natator depressus	Flatback turtle	0.8869	1740
Cnidaria	Anthozoa	Helioporacea	Helioporidae	*Heliopora coerulea*	Blue coral	0.8764	32
		Scleractinia	Acroporidae	*Acropora abrolhosensis*	Acropora	0.9005	20
				Acropora cerealis	Acropora	0.9371	21
				Acropora gemmifera	Acropora	0.9895	22
				Acropora humilis	Acropora	0.9835	33
				Acropora hyacinthus	Acropora	0.8714	20
				Acropora intermedia	Acropora	0.9129	22
				Acropora microphthalma	Acropora	0.9832	20
				Acropora millepora	Acropora	0.8558	22
				Acropora nasuta	Acropora	0.8715	30
				Acropora spicifera	Acropora	0.8693	29
				Acropora tenuis	Acropora	0.826	26
				Astreopora myriophthalma	Startflower coral	0.9761	29
				Isopora brueggemanni	Isopora	0.9924	23
				Isopora palifera	Isopora	0.4924	21
				Montipora grisea	Pore coral	0.9856	21
				Montipora tuberculosa	Hump coral	0.987	25
			Agariciidae	*Coeloseris mayeri*	Tombstone coral	0.8216	28
				Leptoseris mycetoseroides	Leptoseris	0.9987	23
				Pachyseris rugosa	Serpent coral	0.9643	20

				Pachyseris speciosa	Serpent coral	0.5	24
				Pavona explanulata	Pavona	0.995	22
				Pavona varians	Leaf coral	0.9971	39
			Carophylliidae	*Physogyra lichtensteini*		0.9998	20
			Dendrophyllidae	*Turbinaria stellulata*		0.975	20
			Faviidae	*Cyphastrea chalcidicum*	Favid	0.93	22
				Cyphastrea microphthalma	Favid	0.9558	37
				Echinopora lamellosa	Favid	0.9838	37
				Favia matthaii	Favid	0.9822	36
				Favia pallida	Favid	0.9005	32
				Favia speciosa	Favid	0.9832	25
				Favia stelligera	Favid	0.9787	30
				Favites abdita	Favid	0.9814	31
				Favites complanata	Favid	0.9986	27
				Favites pentagona	Favid	0.9875	23
				Favites russelli	Favid	0.9995	26
				Goniastrea edwardsi	Favid	0.9826	22
				Goniastrea pectinata	Favid	0.9956	37
				Goniastrea retiformis	Favid	0.9936	34
				Leptastrea pruinosa	Favid	0.914	22
				Leptastrea purpurea	Favid	0.4588	22
				Leptastrea transversa	Favid	0.9967	27
				Leptoria phrygia	Favid	0.4999	21
				Montastrea curta	Favid	0.905	25
				Montastrea magnistellata	Favid	0.937	27
				Platygyra pini	Lesser valley coral	0.969	33
			Fungiidae	*Fungia concinna*	Fungia	0.9819	20
				Fungia fungites	Common mushroom coral	0.9815	23
				Fungia horrida	Mushroom coral	0.9977	23
				Lithophyllon undulatum	Stone leaf coral	0.4992	20
			Merulinidae	*Merulina scabricula*		0.997	24
			Mussidae	*Lobophyllia hemprichii*	Largebrain root coral	0.9864	33
			Oculinidae	*Galaxea astreata*	Galaxea	0.9907	26
				Galaxea fascicularis	Galaxea	0.9714	39
			Pectiniidae	*Pectinia alcicornis*		0.4972	20
			Pocilloporidae	*Pocillopora damicornis*	Cauliflower coral	0.4936	33
				Pocillopora eydouxi	Cauliflower coral	0.918	20
				Pocillopora verrucosa	Cauliflower coral	0.7453	29
				Seriatopora hystrix	Bush coral	0.6804	28
				Stylophora pistillata	Hood coral	0.9928	28
			Poritidae	*Porites cylindrica*	Hump coral	0.983	27
				Porites lobata	Hump coral	0.9838	24
				Porites vaughani	Hump coral	0.7491	27
			Thamnastriidae	*Psammocora profundacella*		0.6661	31
Echinodermata	Asteroidea	Spinulosida	Echinasteridae	*Echinaster luzonicus*		0.9986	43
		Valvatida	Ophidiasteridae	*Fromia indica*		0.9943	20
				Fromia monilis		0.9716	38
				Linckia laevigata	Blue seastar	0.997	47
				Linckia multifora		0.918	43
				Nardoa tuberculata		0.914	20
			Oreasteridae	*Culcita novaeguineae*		0.907	32
	Echinoidea	Camarodonta	Echinometridae	*Echinometra mathaei*		0.9324	53
				Echinostrephus molaris		0.9967	31
				Heterocentrotus mammillatus		0.9888	23
		Cidaroida	Cidaridae	*Eucidaris metularia*		0.9996	29
		Diadematoida	Diadematidae	*Echinothrix calamaris*		0.9659	23
				Echinothrix diadema		0.9938	24
	Holothuroidea	Aspidochirotida	Holothuriidae	*Bohadschia argus*	Tigerfish sea cumcumber	0.928	36
				Holothuria atra	Lollyfish cucumber	0.997	37
				Holothuria edulis	Pinkfish seacucumber	0.9807	38
				Pearsonothuria graeffei		0.9945	20
	Ophiuroidea	Ophiurida	Ophiocomidae	*Ophiocoma erinaceus*		0.9986	29
Mollusca	Bivalvia	Arcoida	Arcidae	*Arca avellana*		0.8399	38
				Arca ventricosa	Flying-Bird Ark	0.9335	51
				Barbatia amygdalumtostum	Truncated Ark	0.9721	60
				Barbatia plicata		0.9935	23
		Limoida	Limidae	*Lima lima*		0.9266	37
		Mytiloida	Mytilidae	*Modiolus philippinarum*	File shell	0.989	20
				Septifer bilocularis	Philippine horse mussel	0.9904	79
		Pectinoida	Pectinidae	*Chlamys squamosa*	Deck mussel	0.9895	25

			Laevichlamys cuneata		0.9256	20
			Pedum spondyloideum		0.9952	27
	Pterioida	Isognomonidae	*Isognomon isognomum*	Coral boring pearl shell	0.9981	49
		Ostreidae	*Saccostrea cucullata*	Elongate tooth pearl shell	0.9798	49
		Pinnidae	*Streptopinna saccata*	Coral rock oyster	0.994	23
		Pteriidae	*Pinctada albina*		0.987	20
			Pinctada margaritifera		0.9911	58
			Pinna deltodes	Black lip pearl shell	0.9031	28
			Pteria penguin	Deltoid razor clam	0.9712	30
	Unionoida	Carditidae	*Cardita variegata*	Blank banded winged pearl shell	0.9491	48
		Gryphaeidae	*Hyotissa hyotis*		0.5	20
	Veneroida	Cardiidae	*Acrosterigma alternatum*	Flattened oyster	0.9787	25
			Acrosterigma orbitum		0.9814	23
			Acrosterigma reeveanum		0.9986	28
			Fragum fragum		0.9873	22
			Hippopus hippopus	Saw toothed heart cockle	0.9933	46
			Tridacna crocea	Bear paw	0.9818	71
			Tridacna maxima	Boring clam	0.9855	69
			Tridacna squamosa	Burrowing clam	0.9753	76
		Lucinidae	*Codakia punctata*	Fluted clam	0.9706	20
		Mesodesmatidae	*Atactodea striata*	Punctate Codakia	0.9848	23
		Psammobiidae	*Asaphis violaceans*	Triangle shell	0.9762	33
		Tellinidae	*Tellina staurella*	Pacific Asaphis	0.9225	29
		Veneridae	*Gafrarium tumidum*	Cross tellen	0.9992	23
			Lioconcha fastigiata	Swollen circe	0.9929	20
			Periglypta resticulata		0.9549	20
			Periglypta reticulata		0.9151	20
Gastropoda	Cerithimorpha	Cerithiidae	*Cerithium echinatum*	Ara nunome	0.9549	44
			Cerithium nodulosum	Collumn creeper	0.9828	23
			Cerithium novaehollandiae	Giant nodulose creeper	0.9536	52
			Clypeomorus batillariaeformis	Creeper	0.9759	34
			Clypeomorus bifasciata		0.9646	38
			Pseudovertagus aluco		0.9099	23
			Rhinoclavis aspera	Aluco creeper	0.9523	30
			Rhinoclavis brettinghami	Rough creeper	0.9948	29
			Rhinoclavis sinensis	Beautiful creeper	0.9928	32
		Planaxidae	*Planaxis sulcatus*	Obelise creeper	0.9813	39
		Potamididae	*Cerithidea cingulata*		0.8352	28
			Terebralia palustris	Creeper	0.9968	20
	Hypsogastropoda	Buccinidae	*Cantharus fumosus*	Giant mud creeper	0.9712	35
			Cantharus undosus		0.964	23
			Engina concinna		0.9854	21
			Peristernia incarnata		0.9965	48
		Cassidae	*Casmaria erinaceus*		0.9815	28
		Columbellidae	*Pyrene testudinaria*	Hina zuru	0.971	31
			Pyrene varians	Tylers dove shell	0.9726	25
		Conidae	*Conus capitaneus*		0.9293	35
			Conus coronatus	The captain cone	0.8768	34
			Conus distans	Coronated cone	0.9674	20
			Conus ebraeus	Distantly lineated cone	0.9989	35
			Conus lividus	Black and white cone	0.8602	28
			Conus marmoreus	Livid cone	0.8949	20
			Conus miles	Marble cone	0.9349	56
			Conus miliaris	Mile cone	0.9349	44
			Conus monachus	Military cone	0.9769	43
			Conus musicus		0.9809	35
			Conus mustelinus	Music cone	0.9095	20
			Conus pulicarius	Ermine cone	0.9394	27
			Conus rattus	Flea bitten cone	0.8563	20
			Conus sponsalis	Rat cone	0.8882	26
			Conus striatus	Bridal cone	0.8563	20
			Conus terebra	Striated cone	0.8563	24
			Conus textile	Bachelor cone	0.9431	25
			Conus victoriae	Textile cone	0.9957	20
		Costellariidae	*Vexillum vulpecula*	Queen Victorias cone	0.9633	22
		Cypraeidae	*Cypraea annulus*	Gruners mitre	0.9805	22
			Cypraea caputserpentis	Ringed cowry	0.9438	25
			Cypraea carneola		0.9685	31
			Cypraea cylindrica	Purple mouthed cowry	0.9383	34

		Cypraea erosa	Tapering cowry	0.9776	37
		Cypraea errones		0.977	29
		Cypraea isabella	Erroneus cowry	0.953	41
		Cypraea lynx	Fawn coloured cowry	0.9686	26
		Cypraea moneta	Lynx cowry	0.9353	29
		Cypraea quadrimaculata	Money cowry	0.9733	25
		Cypraea tigris	Palish cowry	0.9943	37
	Fasciolariidae	*Latirolagena smaragdula*	Tiger cowry	0.9961	28
		Latirus nodatus	Maru nishi	0.9982	29
		Latirus turritus		0.5	32
		Peristernia fastigium	Botto tsunomata	0.9015	20
		Peristernia nassatula		0.9976	33
		Pleuroploca filamentosa		0.8992	37
	Littorinidae	*Littoraria filosa*	Filamented spindle shell	0.9961	23
		Nodilittorina pyramidalis	Thin periwinkle	0.966	23
		Tectarius rusticus		0.9059	23
	Muricidae	*Chicoreus brunneus*		0.9766	32
		Chicoreus microphyllus	The burnt murex	0.93	24
		Coralliophila neritoidea		0.8222	29
		Cronia aurantiaca	Violet purpura	0.9927	21
		Cronia avellana	Pseudo almond purple	0.9318	37
		Drupa grossularia	Filbert nut buccinum	0.9861	24
		Drupa ricinus	Finger drupa	0.9844	38
		Drupa rubusidaeus	Prickly drupe	0.9977	35
		Drupella cornus	Porcupine castor bean	0.9907	71
		Drupella rugosa	Dogwood drupe	0.9996	24
		Ergalatax margariticola	Hime shiro reishe damashi	0.8764	58
		Morula biconica	Shouldered castor bean	0.9985	32
		Morula granulata	Rough castor bean	0.9958	35
		Morula margariticola	Granulated drupe	0.9775	35
		Morula spinosa		0.9409	50
		Thais aculeata		0.9006	26
		Thais kieneri		0.9988	30
	Nassariidae	*Nassarius glans*	Kieners purple	0.9967	20
	Olividae	*Oliva annulata*	Acorn dog whelk	0.9618	23
	Strombidae	*Lambis chiragra*	Ringed olive	0.9968	35
		Lambis lambis		0.9929	49
		Strombus lentiginosus	Common spider shell	0.9763	26
		Strombus mutabilis	Freckled Stromb	0.8742	20
		Strombus urceus	Flowery stromb	0.9885	57
	Terebridae	*Terebra affinis*	Little bear stromb	0.9709	20
		Terebra maculata		0.9912	24
	Triviidae	*Trivia oryza*	Crenulated auger	0.9926	20
	Turbinellidae	*Vasum ceramicum*	Rice grain bean cowry	0.9859	32
		Vasum turbinellum		0.9889	51
	Volutidae	*Melo amphora*		0.975	32
Nacellina	Lottidae	*Patelloida saccharina*	Baler melon shell	0.9686	32
Neotaenioglossa	Haliotidae	*Haliotis asinina*		0.967	31
		Haliotis ovina	Asss ear	0.9933	20
	Haliotidae	*Haliotis squamata*	Sheep ear shell	0.4931	26
		Haliotis varia	Scaly abalone	0.9854	56
	Trochidae	*Calthalotia strigata*	Variable abalone	0.9873	23
		Clanculus atropurpureus		0.9936	29
		Monodonta labio		0.998	44
		Tectus fenestratus	Lipped periwinkle	0.9842	36
		Tectus niloticus	Latticed top shell	0.9912	36
		Tectus pyramis	Button shell	0.9784	98
		Trochus hanleyanus	Pyramid trochus	0.9668	54
		Trochus histrio	Hanleys trochus	0.9876	34
		Trochus maculatus		0.9957	29
	Turbinidae	*Angaria delphinus*	Maculated top shell	0.9773	53
		Astraea rhodostoma	Lacinate dolphin shell	0.9939	22
		Astralium rotularia		0.9584	54
		Turbo argyrostomus	Knob star shell	0.9748	44
		Turbo chrysostomus		0.997	35
		Turbo cinereus		0.9806	41
		Turbo foliaceus	Moon turban	0.9872	35
Neritopsina	Neritidae	*Nerita balteata*		0.9987	21
		Nerita polita		0.9901	36
		Nerita reticulata	Ancient nerite	0.9672	46
		Nerita undata		0.9892	60

	Nudipleura	Phyllidiidae	*Phyllidia coelestis*	Wavy nerite	0.9392	20
			Phyllidia elegans		0.9629	20
			Phyllidiella pustulosa		0.978	23
	Patellina	Patellidae	*Patella flexuosa*		0.9906	36
Polyplacophora	Neoloricata	Chitonidae	*Acanthopleura gemmata*		0.9688	55
			Acanthopleura spinosa	Northern spined chiton	0.9714	40
